# Characterisation of *Itersonilia* spp. from Parsnip and Other Hosts

**DOI:** 10.3390/jof10120873

**Published:** 2024-12-16

**Authors:** Lauren H. K. Chappell, Guy C. Barker, John P. Clarkson

**Affiliations:** Warwick Crop Centre, School of Life Sciences, Wellesbourne Campus, University of Warwick, Warwick CV35 9EF, UK; guy.barker@warwick.ac.uk (G.C.B.); john.clarkson@warwick.ac.uk (J.P.C.)

**Keywords:** *Itersonilia*, *Pastinaca sativa*, plant pathogen, temperature-dependent growth, phylogenetics, basidiomycete

## Abstract

Parsnips (*Pastinaca sativa*) are a speciality UK crop with an economic value of at least 31M GBP annually. Currently, the major constraints to production are losses associated with root canker disease due to a range of fungal pathogens, among which *Itersonilia pastinacae* is of most concern to growers. With limited research conducted on this species, this work aimed to provide a much-needed characterisation of isolates from across the UK, continental Europe, and New Zealand. Previously, up to four separate *Itersonilia* species have been proposed based on the formation of chlamydospores and host specificity: *I. pastinacae*, *I. perplexans*, *I. pyriformans*, and *I. pannonica*. However, *Itersonilia* spp. isolates principally from parsnip, but also from a range of other hosts, which were found to infect both parsnip roots and leaves in pathogenicity tests. In growth rate assays, isolates were found to grow at temperatures of 0–25 °C and produce both chlamydospores and ballistospores across the same range of temperatures, although chlamydospore production was found to decrease as temperature increased. Following whole genome sequencing, specific primers were designed for the molecular characterisation of the isolates using six housekeeping genes and three highly variable functional genes. Phylogenetic analysis separated isolates into two and six clades, respectively, but the grouping was not associated with hosts or locations. Based on the results of this research, there was no evidence to support more than a single species of *Itersonilia* among the isolates studied.

## 1. Introduction

Parsnip (*Pastinaca sativa*) is a speciality crop with approximately 4000 ha being grown across the UK and an annual economic value of 64M GBP. Root canker disease due to a range of fungal pathogens is the primary cause of economic damage, and, among those implicated, growers show the greatest levels of concern for *Itersonilia pastinacae*. However, despite the wide host range and distribution of *Itersonilia* spp., little research has been conducted on *I. pastinacae*, particularly in relation to ce on parsnip and phylogeny. This paper reports on the molecular and biological characterisation of *Itersonilia* isolates from parsnips and other plant hosts for the first time.

*Itersonilia* spp. are basidiomycetes from the order Cystofilobasidiales, a group consisting primarily of yeasts from cold climates [1]. These species grow in nature as dikaryotic hyphae, but in laboratory cultures, they can develop as monokaryotic hyphae with conidia appearing after an initial phase of mycelial growth [2]. Originally described by Derx [3], the genus comprised only one species, *I. perplexans* Derx., with a second species, *I. pyriformans*, added by Nyland [4], although these two species were soon considered to be co-specific [5]. A third species, *I. pastinacae,* was then described by Channon [6] following isolation from diseased parsnip tissue, whilst a final species, *I. pannonica*, (originally *Udeniomyces pannonicus* [7]), was a more recent discovery following isolation from a range of plants including *Angelica sylvestris* (wild celery) [8].

It was suggested that a major difference between *I. perplexans* and *I. pastinacae* was the presence of thick-walled chlamydospores only within *I. pastinacae* isolates [5,6,9]. However, Boekhout [10] noted that *I. perplexans* strains isolated from chrysanthemum, parsnip, anemone, and dahlia also all produced chlamydospores, indicating that this was not a reliable method for differentiating between these species. The existence of separate species within *Itersonilia* was further questioned when they were noted to have compatible interspecific mating reactions [2]. The more recent paper describing *I. pannonica* (syn. *U. pannonicus*) places this species close to *I. perplexans* using a molecular approach [7]. There is still a need, however, to apply phylogenetic approaches to specifically examine the relationships among the *Itersonilia* spp.

*Itersonilia* spp. are pathogenic on a number of plant species, including parsnip, carrot umbelliferous herbs, and members of the Asteracae family [8,11,12,13]. On parsnips, *I. pastinacae* was identified as the primary cause of root canker, with dark brown/black lesions appearing on the crown and shoulder of the root during the autumn and winter [6]. Critically, Channon [6] also reported that *I. pastinacae* isolates from parsnip were virulent on parsnip, but that *Itersonilia* isolates from other hosts were not, indicating a second major difference between *I. perplexans* and *I. pastinacae* in addition to the former not producing chlamydospores. As seed-borne pathogens with a distinct foliar cycle, *Itersonilia* spp. also cause symptoms of seedling blight, leaf spots, and necrosis [12,14,15] on parsnip and carrot, as well as on herbs such as dill (*Anethum graveolens*), fennel (*Foeniculum vulgare*), parsley (*Petroselinum crispum*), and coriander (*Coriandrum sativum*) [11,13]. Leaf symptoms appear as areas of brown necrotic lesions surrounded by a green/yellow halo that eventually fall away, leaving an exposed hole in the leaf [12].

*I. pastinacae* can be seed transmitted, leading to the infection of parsnip seedlings and mature plants [16], and further foliar transmission is facilitated by the production of ballistospores, which are forcibly discharged through the formation of Buller’s drop [17]. The infection of foliage results in necrotic lesions, which, in turn, develop further ballistospores, which can coat newly formed seed on flowering parts, hence completing the lifecycle. However, the transmission of *I. pastinacae* to parsnip roots is not fully understood, although one theory proposes that necrotic lesions from the infected foliage drop onto the exposed crown and shoulder of the developing roots in the soil, leading to the development of cankers [16,18]. Similarly, Channon [12] also suggested that lesions on infected parsnip leaves provide ballistospores not only for airborne dissemination, but also for root infection, while Smith [19] reported that chlamydospores enable the fungus to survive in soil. Hence, the role of *I. pastinacae* chlamydospores in relation to ballistospores in initiating cankers has yet to be clearly defined. In culture, *Itersonilia* spp. has demonstrated mycelial growth at 19–20 °C [17]; however, given its relatedness to cold yeasts, which have the ability to grow at 0 °C [1], and the survival potential of chlamydospores in soil [19], it can be deduced that the full temperature range of *Itersonilia* spp. is yet to be determined.

Since the work of Channon in the 1960s, there has been little further research on *I. pastinacae*, and no studies investigating the molecular and biological diversity of *Itersonilia* spp. from parsnip and other hosts have been conducted. Little is known about variations in virulence between and within *Itersonilia* spp. and effect of temperature on development. Therefore, the primary objective of this work was to describe the biological characteristics of *Itersonilia* spp. isolates using pathogenicity assays and the effect of temperature on mycelial growth and spore production. A second aim was to conduct a molecular characterisation of *Itersonilia* spp. for the first time through housekeeping and functional gene analysis. The focus of this work was on isolates from parsnip, but for comparative purposes, a smaller collection of isolates was obtained from other hosts (chrysanthemum, dill, fennel, and parsley). These data provide further insight into the question of *Itersonilia* speciation.

## 2. Materials and Methods

### 2.1. Collection of Itersonilia Isolates

A total of 51 *Itersonilia* spp. isolates were collected from various locations and hosts (Table 1), with the majority from parsnip (42 isolates), but others from chrysanthemum (3), dill (4), fennel (1), and parsley (1). For the purposes of this paper, based on existing literature, all isolates derived from parsnip will be provisionally referred to as *I. pastinacae* and those from other hosts as *Itersonilia* sp. Cultures were isolated from the infected parsnip roots, foliage, seeds (42 isolates), and foliage of other host species (9 isolates) using the suspension method [20,21], whereby infected tissue is attached to the lid of a Petri dish and *Itersonilia* spp. ballistospores are discharged onto the agar. Four 5 mm^2^ pieces of infected tissue or individual parsnip seeds were attached to the lids of Petri dishes with Vaseline (Sigma-Aldrich, Gillingham, UK), suspended over malt agar 2% (MA), and incubated for 5–10 days at 20 °C. Once mycelial growth was evident, fungal colonies were subcultured onto MA and incubated for 5–10 days at 20 °C. Isolates were stored on 0.2% MA and slopes at 4 °C or as frozen mycelial MA (0.2%) plugs in Potato Dextrose Broth (PDB; Sigma-Aldrich, Gillingham, UK) amended with 20% glycerol at −20 °C.

### 2.2. Virulence of Itersonilia spp. Isolates on Parsnip Roots

Experiments were conducted to assess the virulence of 48 *Itersonilia* spp. isolates (only DNA was available for IP14 [chrysanthemum], IP16 [dill], and IP18 [parsnip]; Table 1) on freshly harvested parsnip roots (cv. Picador) from a commercial grower. Roots were mechanically harvested, washed with tap water, surface sterilized with 70% ethanol (*v*/*v*), and air-dried. Each parsnip was checked for any disease symptoms before the widest part of the root was inoculated with a 5 mm agar plug of each *Itersonilia* spp. isolate taken from the leading edge of a 12-day-old actively growing colony. Parsnips were then incubated in the dark at 12 °C on damp tissue in transparent clip-sealed 3 L plastic boxes (Sistema, Auckland, New Zealand), and moist conditions were maintained to encourage disease development by misting roots with sterile RO water once a week. Six replicate parsnip roots were established for each *Itersonilia* spp. isolate, and three repeat experiments were conducted. Control roots were prepared as described above and inoculated with a 5 mm plug of malt agar; roots were monitored for any disease development. Photographs of symptoms were taken weekly from three weeks post inoculation, and the lesion area was measured using ImageJ [22]. Differences in lesion size between isolates were assessed using a one-way ANOVA implemented in R (version 0.98.945, R Development Core Team, Vienna, Austria, 2014). Preliminary experiments were conducted to determine optimum inoculation conditions, following which, the re-isolation of *Itersonilia* spp. From parsnip roots was conducted to confirm pathogen identity and fulfil the requirements of Koch’s postulates.

### 2.3. Virulence of Itersonilia spp. Isolates on Detached Parsnip Leaves

The same set of 48 *Itersonilia* isolates (Table 1) was used to assess virulence on detached parsnip leaves. Parsnip plants (cv. Panache) were grown from seed in a 2:1 mix of Levington F2 compost (BHGS, Evesham, UK) and sharp sand (BHGS, Evesham, UK) in 2 L pots and placed in a glasshouse compartment at 20 °C. When the plants were sixteen weeks old, leaves were removed for inoculation. Ballistospore suspensions of *Itersonilia* isolates were produced by adding 1 mL sterile RO water to 14-day-old cultures, rubbing with a spreader and filtering through a milk filter (190 mm; Goat Nutrition Ltd., Ashford, UK) to remove mycelium. Spore numbers were determined using a haemocytometer and adjusted to 1 × 10^5^ spores mL^−1^. Detached parsnip leaves were placed into 1 L clip-sealed plastic boxes (Sistema, Auckland, New Zealand) on damp tissue and inoculated with 2 × 20 μL drops of spore suspension on each side of the central vein, with water-only control also established with 2 × 20 uL drops of RO water. The boxes were clip-sealed and placed in a controlled environment at 20 °C with a 16 h photoperiod under white fluorescent bulbs. The boxes were removed after seven days, and photographs of the lesions were taken. The lesion area was then measured using ImageJ [22]. There were twelve detached leaves inoculated for each *Itersonilia* spp. Isolate, and four repeat experiments were conducted. Differences in lesion size between isolates were assessed using a one-way ANOVA on log_e_-transformed data.

### 2.4. Effect of Temperature on Itersonilia spp. Growth Rate

The set of 48 *Itersonilia* isolates (Table 1) was also used to determine the effect of temperature on mycelial growth rate on MA. Petri dishes containing 25 mL of MA were centrally inoculated with a 5 mm mycelial plug taken from the leading edge of a 12-day-old actively growing culture of each *Itersonilia* sp. isolate. Plates were sealed with parafilm and incubated at 0, 5, 10, 15, 20, and 25 °C, and a total of four replicate plates per isolate were prepared for each temperature. Measurements of the colony diameter at two perpendicular points were recorded twice weekly over 30 days, and the growth rate was calculated (mm day^−1^). Following these experiments, *I. pastinacae* isolate IP10 was selected as a standard isolate to test the effect of additional temperatures of 2.5, 17.5, 22.5, 27.5, and 30 °C. As before, there were four replicate plates per temperature with twice weekly measurements of growth for 30 days. A Briere curve was fitted to the data, modified to accommodate the growth rate at temperatures close to 0 °C [23]. Differences between isolates were assessed using a one-way ANOVA with a post hoc ‘Tukey’ test to identify differences at each temperature.

### 2.5. Effect of Temperature on Itersonilia spp. Spore Production

The chlamydospore and ballistospore production of the 48 *Itersonilia* spp. isolates was determined at different temperatures. A 5 mm mycelial plug from the leading edge of a 12-day-old actively growing culture of each *Itersonilia* sp. isolate was placed in the centres of Petri dishes containing 25 mL MA. The plates were incubated at 0, 5, 10, 15, 20, and 25 °C in darkness for 14 days, after which, 1 mL sterile RO water was added, and spores were gently removed from the fungal colonies using a sterile spreader. The spore suspension was filtered through a milk filter (Goat Nutrition Ltd., Ashford, UK) to remove mycelium, and the number of chlamydospores and ballistospores was determined using a haemocytometer. Four replicate plates were established per isolate at each temperature, with ten haemocytometer counts per Petri dish. The mean log_10_ spore count density (spores mm^−2^ of colony) for each spore type was analysed using a one-way ANOVA, and a post hoc ‘Tukey’ was applied to determine differences between isolates at each temperature.

### 2.6. Genome Sequencing and Assembly of I. pastinacae

*I. pastinacae* isolate IP10 (Table 1) was grown in Petri dishes containing 25 mL PDB and incubated at 20 °C for seven days, after which, fungal mycelium was harvested and lyophilized overnight. Genomic DNA was extracted from the freeze-dried mycelium using the DNeasy Plant Mini Kit (Qiagen Ltd., Manchester, UK), using the manufacturer’s protocol. The genomic library was prepared using the Illumina TruSeq DNA library preparation kit following the manufacturer’s protocol (Twist Bioscience, San Francisco, CA, USA) and was sequenced on a HiSeq 2500 (Illumina, Cambridge, UK) using a 51 bp paired end read. De novo assembly of the *Itersonilia* genome was built using SPAdes (Version 3.8.2), and a contig assembly file with an N50 value (the median length of 50% of the assembly contigs) of 1.6 kb was obtained.

### 2.7. Molecular Characterisation of Itersonilia spp. Isolates

DNA from 51 *Itersonilia* isolates (Table 1) was extracted as described for *I. pastinacae* IP10 previously. Species identity and diversity were first investigated through PCR amplification and sequencing of the ITS regions of the ribosomal DNA, as well as regions of 18S ribosomal RNA gene (*SSU*), 28S ribosomal RNA gene (*LSU*), translation elongation factor 1-α (*EF1-*α), RNA polymerase II (*RpbII*), and β-tubulin (*TUB2*). Published universal fungal primers were used for amplifying the *ITS* and EF1-α regions, while primers for other housekeeping genes were designed using the *I. pastinacae* whole genome sequence (Table 2). Due to the lack of genetic variability within the housekeeping genes, further additional functional genes with variable sequence were identified by comparing annotated sequences of the *Cryptococcus* spp. genome with the *I. pastinacae* genome using BLAST analysis on NCBI. Three of these, triosephosphate transporter family (TTF), tRNA methyl transferase (tMT), and cellobiose dehydrogenase (CDH), were selected for PCR amplification based on the predicted genetic variability to investigate intra-species diversity using primers designed from the *I. pastinacae* genome sequence. PCR was conducted in 20 µL reactions consisting of 10 µL 1xREDTaq Ready Mix PCR reaction mix (Sigma-Aldrich, UK), 2 µL DNA template (10 ng), 1 µL per primer template (0.4 µmol L^−1^), and 7 µL purified water (Sigma-Aldrich, UK). Specific annealing temperatures and thermocycling conditions were used for each primer pair (Table 2). Following PCR, amplicons were visualized using gel electrophoresis, purified using the QIAquick PCR purification Kit (Qiagen, UK) and submitted for sequencing by GATC (Konstanz, Germany). Gene sequences were trimmed and aligned using the ClustalW algorithm and refined using the MUSCLE algorithm implemented in MEGA v6 [24]. A multi-locus concatenated phylogenetic tree was constructed using the ‘Maximum Likelihood’ option, rooted using *Cystofilobasidiales macerans* (GenBank Genome GCA_014825765.1) [25].

## 3. Results

### 3.1. Virulence of Itersonilia spp. Isolates on Parsnip Roots

**Figure 1:**
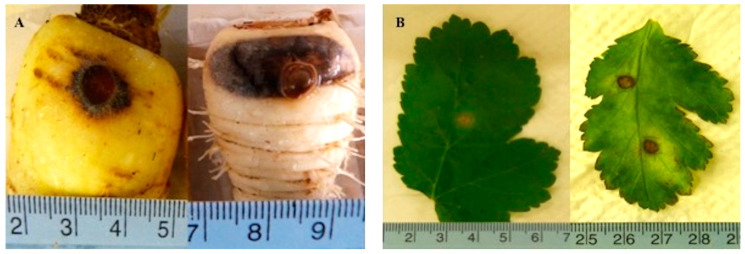
(**A**) Symptoms of *Itersonilia* on inoculated parsnip roots (cv. Picador) after 21 days at 20 °C. Left: weakly virulent *Itersonilia* isolate IP15. Right: highly virulent *Itersonilia* isolate IP50. (**B**) Symptoms of *Itersonilia* on inoculated parsnip leaves (cv. Panache) after 7 days at 20 °C. Left: weakly virulent isolate IP8. Right: highly virulent isolate IP47.

**Figure 2:**
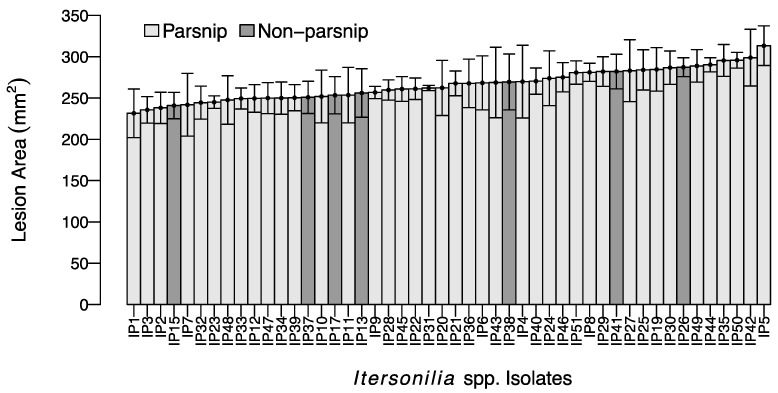
Mean lesion size (mm^2^) on parsnip roots (cv. Picador) for 48 different *Itersonilia* isolates. Error bars represent the standard error of the mean (SEM) for four independent replicates. Light grey bars represent isolates from parsnip hosts; dark grey indicates isolates from non-parsnip hosts (chrysanthemum, dill, fennel, and parsley [Table 1]).

Following inoculation with *Itersonilia* isolates, all parsnip roots developed typical black canker lesions (Figure 1A), with no lesions developing on the control roots. The mean lesion area ranged from 220 mm^2^ to 320 mm^2^ after 60 days (Figure 2). There was continuous variation in lesion area across the *Itersonilia* isolates, but no groups or outliers were identified within the dataset. The ANOVA of the lesion area (cm^2^) indicated that there was no significant effect of *Itersonilia* isolate on lesion size (*p* > 0.05). *Itersonilia* isolates from parsnip, chrysanthemum, dill, fennel, and parsley were evenly distributed, with no detectable correlation between the host origin and lesion area on the parsnip. Re-isolation from the necrotic lesions confirmed *Itersonilia* spp. as the causal agent, fulfilling the requirements of Koch’s postulates.

### 3.2. Virulence of Itersonilia spp. Isolates on Detached Parsnip Leaves

**Figure 3:**
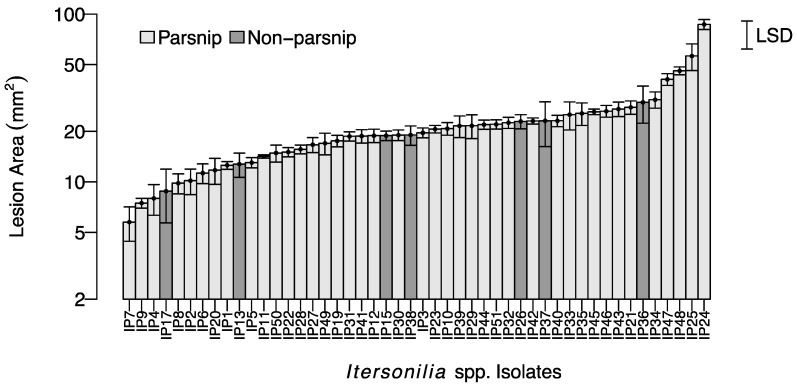
Mean lesion size (mm^2^) on detached parsnip leaves (cv. Panache) for 48 different *Itersonilia* isolates. Data are plotted on a log scale; error bars represent the SEM for four independent replicates. LSD is indicated at the 5% level. Light grey bars indicate isolates from parsnip hosts; dark grey bars indicate isolates from non-parsnip hosts (chrysanthemum, dill, fennel, and parsley [Table 1]).

Following inoculation with *Itersonilia* isolates, necrotic lesions developed on detached parsnip leaves (Figure 1B), with the mean lesion area ranging from 4 mm^2^ (IP7) to 90 mm^2^ (IP24; Figure 3). The ANOVA analysis of the Log_e_-transformed data showed a significant effect of isolate (*p* < 0.05) on lesion size. Whilst *Itersonilia* spp. isolates from different hosts were evenly distributed, with no correlation between host and lesion area, the post hoc 5% LSD values indicated that IP7 and IP24 from parsnip, which had the largest lesion sizes, were significantly more virulent than all other isolates (*p* < 0.05), while isolates IP25 and IP48 (also from parsnip) were also more virulent than the majority (*p* < 0.05). It was also noted that IP36 from parsley was also highly virulent on parsnip leaves. There was no correlation between the virulence of *Itersonilia* isolates in the leaf and parsnip root tests, suggesting that virulence may be tissue specific. The water-only control leaves showed no sign of infection and remained healthy throughout the assay.

### 3.3. Effect of Temperature on Itersonilia spp. Growth Rate

**Figure 4:**
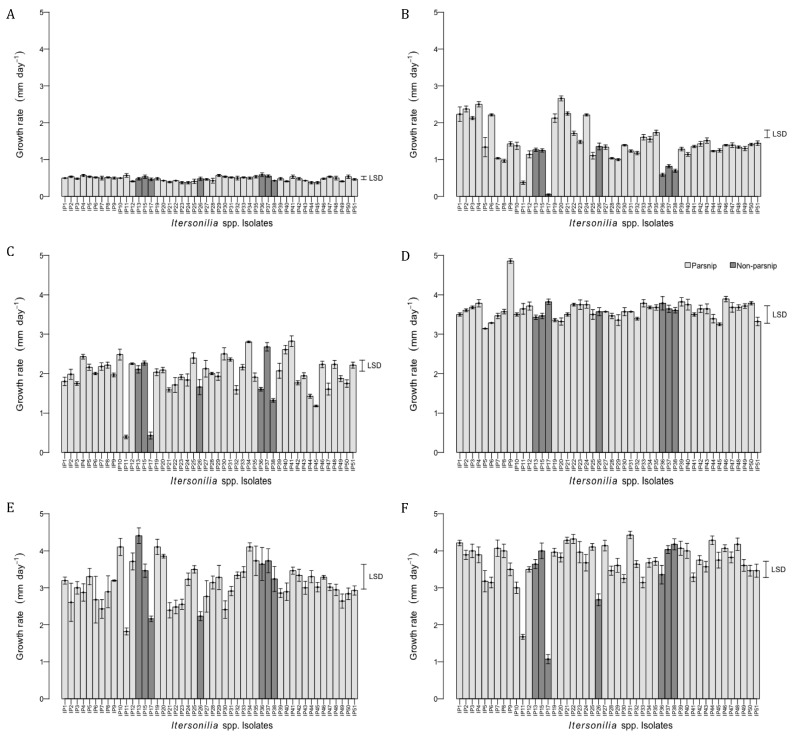
Effect of temperatures (**A**) 0 °C, (**B**) 5 °C, (**C**) 10 °C, (**D**) 15 °C, (**E**) 20 °C, and (**F**) 25 °C on the mean growth rate of *Itersonilia* isolates on MA. Error bars represent the SEM for four independent replicates. LSD is indicated at the 5% level. Light grey bars indicate isolates from parsnip hosts; dark grey bars indicate isolates from non-parsnip hosts (chrysanthemum, dill, fennel, and parsley [Table 1]).

**Figure 5:**
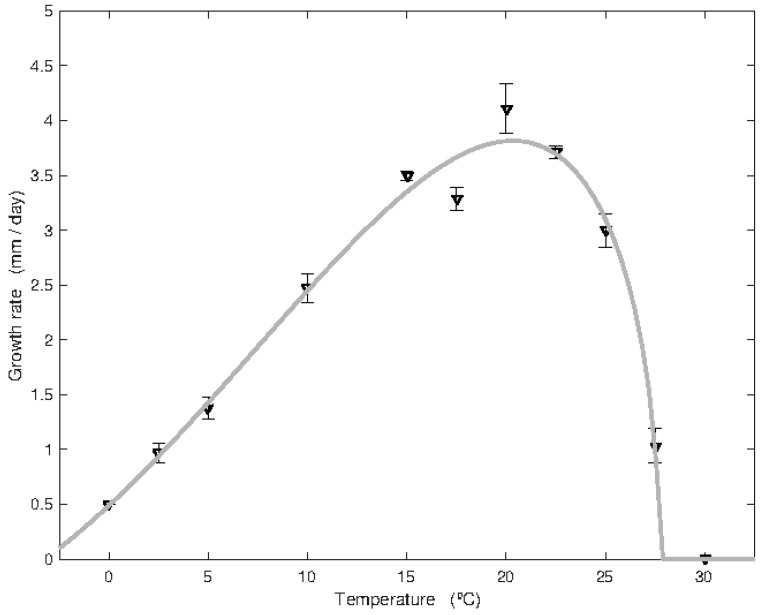
Effect of temperature on the mean growth rate of *I. pastinacae* isolate IP10. Error bars represent the SEM for four independent replicates. The grey line represents a fitted Briere curve.

All *Itersonilia* isolates grew at all six temperatures tested (0, 5, 10, 15, 20, and 25 °C), with a general trend of the growth rate increasing with the temperature over the range (Figure 4). The minimum mean rate of growth over all isolates was at 0 °C (0.48 mm day^−1^), with values ranging from 0.38 to 0.59 mm day^−1^, and the maximum mean growth rate was at 25 °C (3.66 mm day^−1^), with values ranging from 1.07 to 4.43 mm day^−1^. Variation in growth rate between isolates was smallest at 0 °C and greatest at 5 °C (0.05–2.66 mm day^−1^). Some isolates such as IP26, IP45, and particularly IP11 (parsnip) and IP17 (dill) displayed consistently slower growth rates compared to other isolates at most temperatures, whilst isolates IP4, IP34, IP46 (parsnip), and IP37 (dill) consistently exhibited faster growth rates (Figure 4). Some isolates such as IP38 (parsley) and IP44 (parsnip) appeared to grow preferentially at high temperatures, whilst others, including IP2, IP6, and IP30 (parsnip), appeared to grow better at low temperatures. The ANOVA revealed significant differences (*p* < 0.001) between isolate growth rates at each temperature. A post hoc ‘Tukey’ analysis conducted to perform pairwise comparisons between isolates showed that the largest number of highly statistically significant differences (*p* < 0.001) was obtained at 5 °C, while the highest degree of homogeneity was at 0 °C and 20 °C (Figure 4). Isolates IP11 (parsnip) and IP17 (dill) were often identified as outliers, displaying particularly low growth rates at all temperatures other than at 15 °C. In addition to the six temperatures tested above, the growth rate of the genome-sequenced standard isolate IP10 from parsnip was determined for further five temperatures of 2.5, 17.5, 22.5, 27.5, and 30 °C (Figure 5). This isolate displayed the lowest mean rate of growth at 0 °C (0.5 mm/day), with no growth at 30 °C, and a maximum rate of growth between 20 and 22.5 °C (4.11 and 3.71 mm day^−1^, respectively), and was ranked consistently in the middle growth rate range of all the isolates (Figure 4).

### 3.4. Effect of Temperature on Itersonilia spp. Spore Production

**Figure 6:**
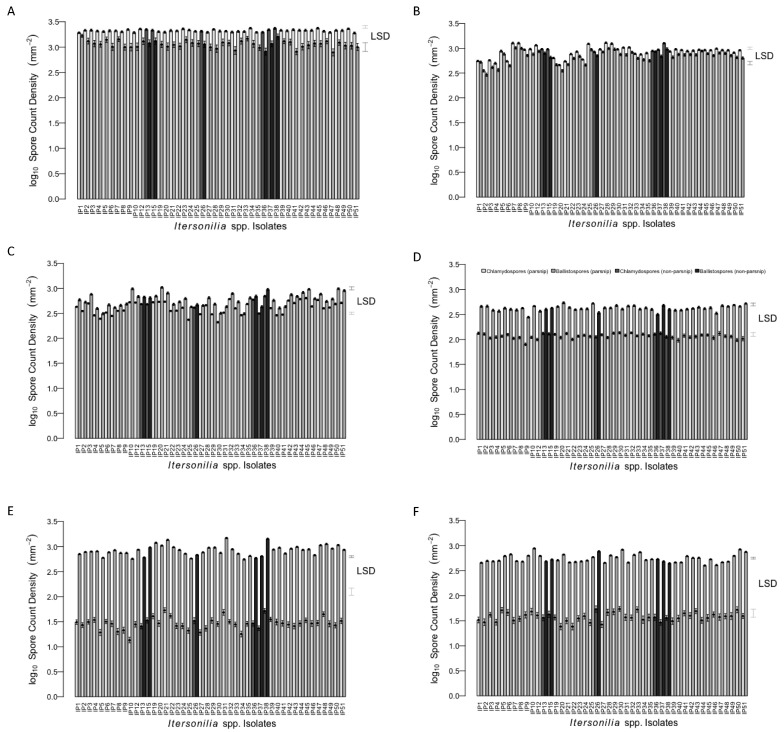
Effect of temperatures (**A**) 0 °C, (**B**) 5 °C, (**C**) 10 °C, (**D**) 15 °C, (**E**) 20 °C, and (**F**) 25 °C on mean log_10_ spore density (spores mm^−2^) for different *Itersonilia* isolates. Data points represent mean spore density for four replicates; error bars represent the SEM for four independent replicates. LSD is indicated at the 5% level. Lighter bars indicate isolates from parsnip hosts; darker bars indicate isolates from non-parsnip hosts (chrysanthemum, dill, fennel and parsley [Table 1]).

Chlamydospores and ballistospores were produced by all Itersonilia isolates over the six temperatures examined (0, 5, 10, 15, 20, and 25 °C), quantified as spore density (log10 spores mm^−2^), to control for variations in colony size observed at different temperatures. The overall trend was for chlamydospores to be produced predominantly at lower temperatures, while ballistospore production was more consistent across the entire temperature range (Figure 6). Ballistospore density ranged from 1.96 to 4.02 log10 spores mm^−2^ across all temperatures and showed a similarly high range of values at both 0 °C (2.90–3.22 log10 spores mm^−2^) and 20 °C (2.75–3.17 log10 spores mm^−2^). The highest mean chlamydospore density was at 0 °C, with values ranging from 3.29 (IP1, parsnip) to 3.38 (IP38, parsley) log10 spores mm^−2^, and decreased steadily with increasing temperature. The lowest mean chlamydospore density across all isolates was at 20 °C, where values ranged from 1.13 (IP10, parsnip) to 1.73 (IP21, parsnip) log_10_ spores mm^−2^. *Itersonilia* isolates IP11 (parsnip) and IP17 (dill) did not produce any spores at any temperature, so they were removed from the graph and subsequent analysis. The ANOVA analysis showed a significant effect of *Itersonilia* isolate (*p* < 0.05) on spore production for both ballistospores and chlamydospores, while the post hoc ‘Tukey’ analysis identified significant differences between isolates for both chlamydospore and ballistospore production across all temperatures (0, 5, 10, 15, 20, and 25 °C). The results showed a broad increase in significant differences between isolates (*p* < 0.05) as the temperature increased, with the largest number of highly statistically significant differences (*p* < 0.001) at 20 °C, while at 0 and 15 °C, isolates showed the highest degree of homogeneity.

### 3.5. Molecular Characterisation of Itersonilia spp. Isolates

**Figure 7:**
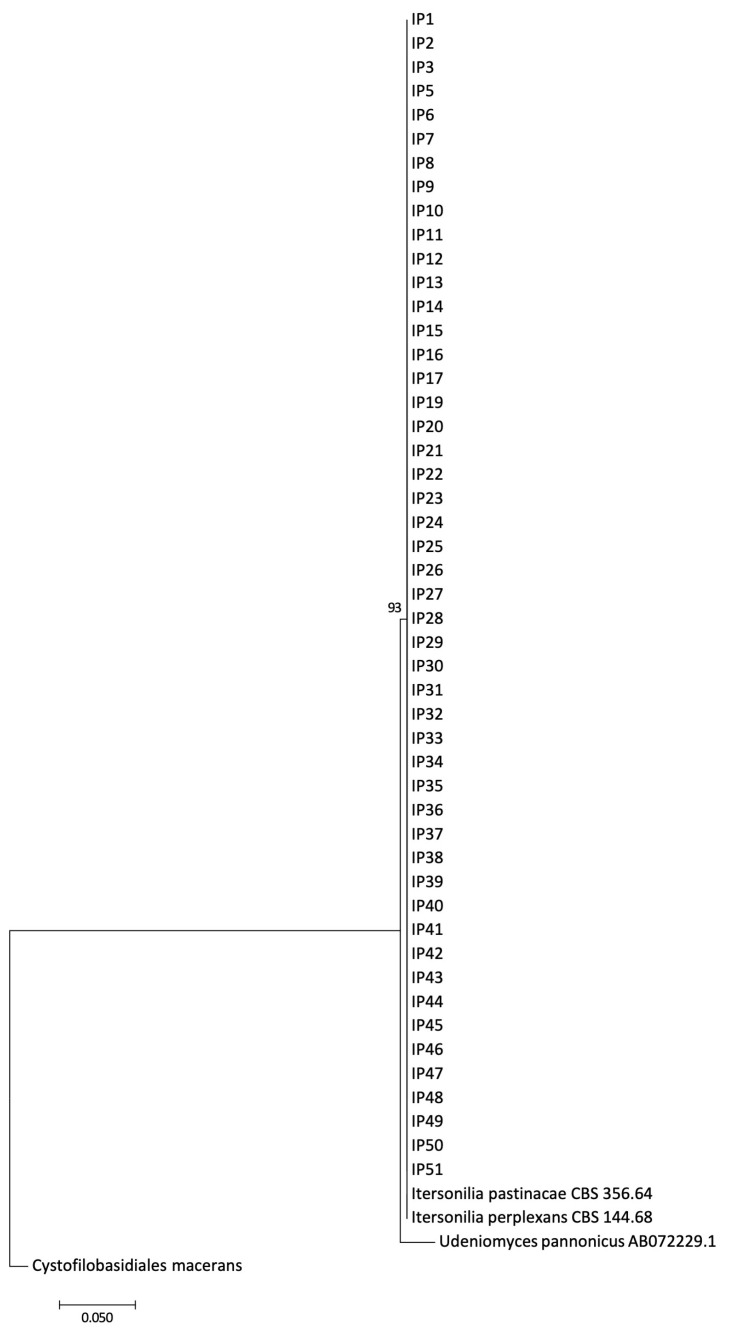
Maximum likelihood phylogenetic tree for *Itersonilia* spp. isolates based on the internal transcribed spacer region (ITS) of the rDNA (GenBank accession numbers MG198712-MG198760) alongside reference isolates for *Itersonilia pastinacae* (GenBank accession number CBS 356.64), *Itersonilia perplexans* (GenBank accession number CBS 144.68), and *Udenomyces pannonicus* (GenBank accession number AB072229.1). Numbers represent bootstrap values from 1000 replicates. Scale bar indicates 0.05 substitutions per site. The tree is rooted through *Cystofilobasidiales macerans* (GenBank Genome GCA_014825765.1) [25]. ITS sequence for an *I. perplexans* reference isolate (IMI 264396) as taxonomically described by Ingold [9] is also included (AB072233.1).

**Figure 8:**
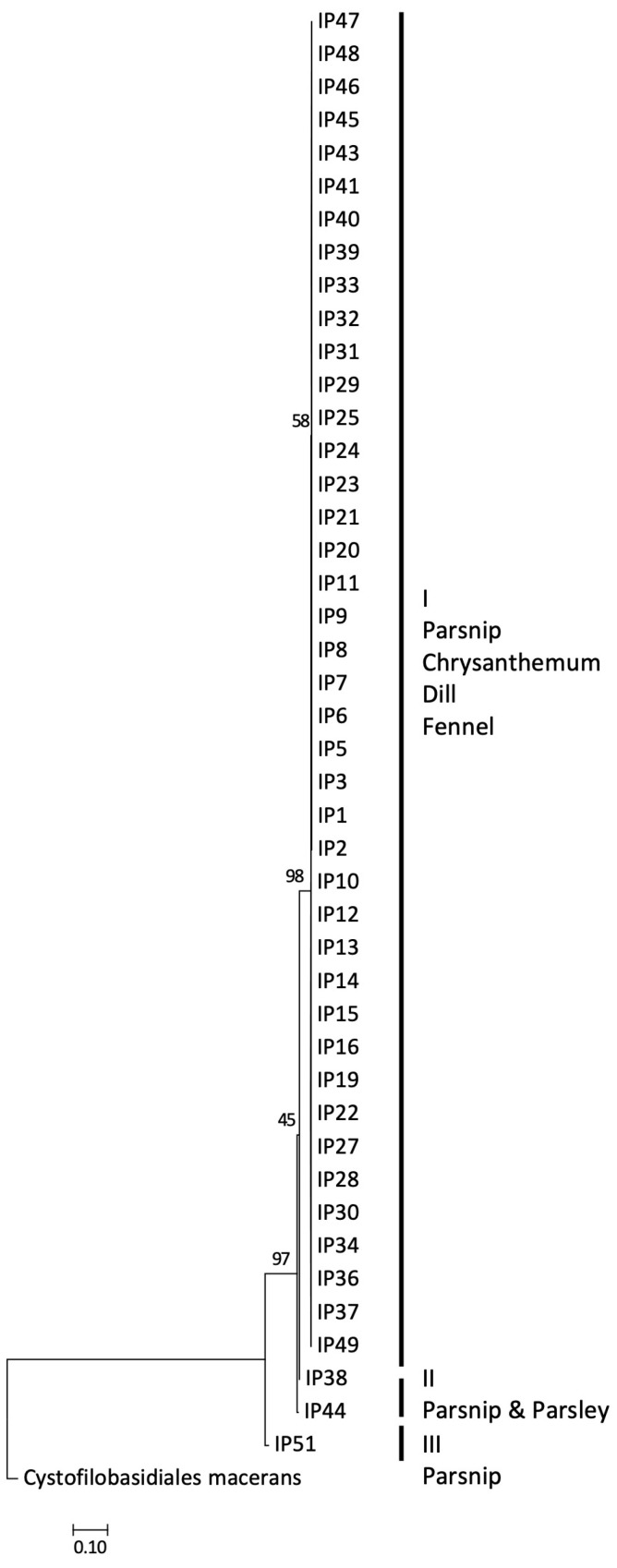
Maximum likelihood phylogenetic tree for *Itersonilia* spp. isolates based on concatenated sequences for the internal transcribed spacer region (ITS) of the rDNA (GenBank accessions MG198712-MG198760), RNA polymerase II (*Rpb-II*), translation elongation factor (*EF-1α*), large ribosomal subunit (*LSU*) (GenBank accessions MG241126-MG241175), small ribosomal subunit (*SSU*) (GenBank accessions MG241176-MG241225), and beta-tubulin (*TUB2*). Numbers represent bootstrap values from 1000 replicates. Scale bar indicates 0.10 substitutions per site. The tree is rooted through *Cystofilobasidiales macerans* (GenBank Genome GCA_014825765.1) [25].

**Figure 9:**
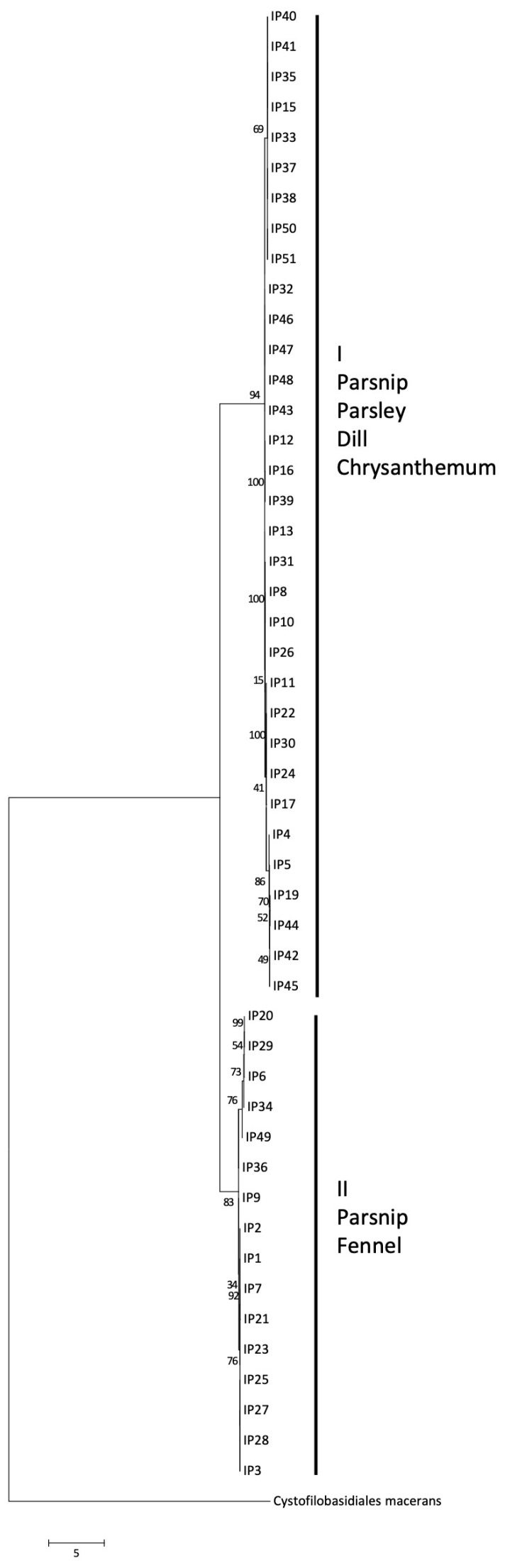
Maximum likelihood phylogenetic tree for *Itersonilia* spp. isolates based on the functional genes triosephosphate transporter family (*TTF*), tRNA methyl transferase (*tMT*), and cellobiose dehydrogenase (*CDH*). Numbers represent bootstrap values from 1000 replicates. Scale bar indicates 5 substitutions per site. The tree is rooted through *Cystofilobasidiales macerans* (GenBank Genome GCA_014825765.1) [25].

Following confirmation of *Itersonilia* species identity through *ITS* sequencing and BLAST analysis, sequence data for *ITS*, *EF-1α*, *Rpb-II*, *LSU*, *SSU,* and *TUB2*, and for the three functional genes TTF, tMT, and CDH, were aligned, and phylogenetic analyses were conducted. The maximum likelihood analysis for the *ITS* locus resulted in a single clade for all *I. perplexans* and *I. pastinacae* isolates, including the type cultures for both species, with an additional clade for the *U. pannonicus* (GenBank sequence; Figure 7). No further divisions were evident, indicating that there was no separation of isolates based on *Itersonilia* species host or geographic origin. Bootstrap values on the maximum likelihood analysis were above 93%, indicating good support for clades. The concatenated alignments of the housekeeping genes *ITS*, *EF-1α*, *Rpb-II*, *LSU*, *SSU,* and *TUB2* analysed using maximum likelihood divided the *Itersonilia* isolates into three main clades (I, II, and III), with one clade (III) further divided into sub clades (Figure 8). Clade I contained a single isolate from parsnip, clade II contained two isolates from parsley and parsnip, and clade III contained all remaining isolates from a range of hosts. None of the isolates within clades or sub clades appeared to be related by host or geographical location, with isolates in a single sub clade being isolated from a range of host species and locations. The bootstrap values for the maximum likelihood analysis ranged from 45–98%, with half branches between above 97%; these high values indicated good support for branches. A maximum likelihood phylogeny based on concatenated alignments of the three functional genes TTF, tMT, and CDH divided the *Itersonilia* isolates into two major clades, with each clade further divided into further sub clades (Figure 9). Each of the smaller sub clades contained between 4 and 10 isolates, none of which appeared to be linked to host or geographical location. The two main clades contained isolates from parsnip, parsley, dill, and chrysanthemum (I) and parsnip and fennel (II), and both clades contained isolates from a range of locations. This again suggested that isolates were not grouped based on host species or geographical origin. The bootstrap values ranged from 34–100%; however, the majority of branches displayed values greater than 70%, indicating good support. All analyses were repeated using the neighbour joining approach, leading to the same conclusions.

## 4. Discussion

This is the first study to examine the diversity and biological characteristics of *Itersonilia* from parsnip since the work of Channon [6,29,30] and also provides the only molecular analysis of isolates from different hosts. A major finding was that all *Itersonilia* isolates, including those from hosts other than parsnip, were pathogenic on parsnip roots. Furthermore, although there were some differences in virulence between isolates on parsnip leaves, there was no clear evidence that isolates from hosts other than parsnip were non-pathogenic or less virulent; for instance, one isolate from parsley resulted in some of the largest lesions in this assay. This is in direct contrast to the work of Channon [6], who reported that the majority of *Itersonilia* isolates from parsnip were pathogenic on both roots (32 of 40 isolates) and leaves (all 21), while none of those from other hosts were pathogenic on parsnip (10 and 14 isolates tested on roots and leaves, respectively). Variation in isolate virulence on leaves could, however, affect the transmission rate of *Itersonilia*. Firstly, larger lesions will result in increased ballistospore production and, hence, greater disease pressure in parsnip foliage. Secondly, if ballistospores are responsible for initiating parsnip root cankers, then root infection will also be increased. However, further work is needed to understand the role of both ballistospores and chlamydospores in initiating *Itersonilia* root infections.

This is also the first study to comprehensively examine the effect of temperature on the growth and sporulation of *Itersonilia*. The only previous work by Ingold [31] focused on a single isolate of *Itersonilia* (IMI 264396 from Fagus) grown on malt agar at 20 °C and reported a radial growth rate of approximately 2 mm per day. By comparison, growth rates between 1.8 and 4.3 mm day^−1^ were observed at 20 °C across the *Itersonilia* isolates tested here (mean 3.1 mm day^−1^). It was also noted that *Itersonilia* isolates could grow at temperatures as low as 0 °C, with significant growth at 5–10 °C and an optimum of between 15 and 25 °C. The majority of fungi do not grow below 5 °C [32], but these results are in accordance with *Itersonilia* being a member of the order Cystofilobasidiales, a group of cold yeasts able to maintain growth at temperatures as low as −12 °C [33,34]. The incidence of *Itersonilia* and parsnip canker has also previously been found to increase in colder conditions, with low temperatures (0 °C) failing to halt the growth or sporulation of *Itersonilia*-infected chrysanthemum inflorescences [6,35]. Parsnips are also a predominantly winter crop and, with the mean winter temperature ranging between −1 °C and 6 °C across the UK, the ability of *Itersonilia* spp. to maintain growth at such low temperatures explains the prevalence of cankers in parsnip crops during prolonged periods of cold. Furthermore, the increase in *Itersonilia* spp. incidence during winter may also be due, in part, to reduced activity and competition from other saprophytic fungi.

Ballistospore production across *Itersonilia* isolates was fairly consistent over the temperature range tested, but chlamydospore production was greatest at 0–10 °C and decreased substantially between 10 and 25 °C. Based on these results, it is likely that ballistospores are more abundant in warmer temperatures during spring and summer and represent the primary infection propagule of parsnip foliage and seed, as reported previously [36]. As temperatures decrease in the autumn, chlamydospores potentially then become more abundant, and we would hypothesise that these may represent the primary infection propagule of parsnip roots. This is supported by the observations that *Itersonilia* chlamydospores prolong the survival of the pathogen and that ballistospores are very quickly lysed upon contact with soil [19]. Infection of roots may also be facilitated during the winter by extensive parsnip leaf die back and the dropping of leaves over the top of the parsnip roots. Although Ingold [37] studied the germination of ballistospores in culture, detailing 70% germination after 6 h at 20 °C in unilateral daylight on malt aga, further work is required to understand germination of both ballistospores and chlamydospores on parsnip tissue.

Two key characteristics have been reported in the literature as discriminating factors between the previously described *Itersonilia* species, *I. perplexans* and *I. pastinacae*. Firstly, *I. pastinacae* isolates obtained from parsnip are non-pathogenic on host species of *I. perplexans*, and secondly, abundant production of chlamydospores occurs only in *I. pastinacae* isolates [5,12,20]. Both of these factors are in direct contrast to the results of this study, where isolates from other hosts, including chrysanthemum, dill, parsley, and fennel, were shown for the first time to infect parsnip roots and leaves. Furthermore, all *Itersonilia* isolates produced both chlamydospores and ballistospores, and there was no difference in relative spore numbers between isolates related to the hosts they were collected from. These biologically based results, therefore, question the concept of these two separate *Itersonilia* species. As morphological distinctions to discriminate species are often unreliable [37,38], we, therefore, additionally performed extensive DNA phylogenetic analyses of *Itersonilia* isolates from different hosts using six housekeeping and three functional genes for the first time, which further support these conclusions. In both the housekeeping and functional gene phylogenies, only one clade was comprised solely of isolates from parsnip, while all the other clades included isolates from a variety of the other hosts (chrysanthemum, dill, fennel, and parsley). Clades also often contained isolates from different geographical locations, suggesting widespread distribution, as evidenced by *Itersonilia* being identified not only in a range of agricultural hosts, but also in a wide range of different environmental samples [39,40]. However, although we have demonstrated that *Itersonilia* isolates from dill, fennel, parsley, and chrysanthemum can infect parsnip, further reciprocal work is still required to demonstrate that parsnip isolates can also infect a range of other hosts with no consistent reduction in virulence. Also, given the small number of isolates from other hosts included in this research, further work incorporating a higher number of isolates from outside Europe and other hosts is required.

Further knowledge gained in this study has some important implications for disease control. Firstly, as *Itersonilia* can affect a range of hosts including weed species [35], and isolates in this study all produced ballistospores over a wide temperature range, regular use of fungicides will be required to reduce infection and minimise seed contamination. Detection of ballistospore inoculum, as first investigated by Channon [12], would aid in the effective timing of fungicide applications, and more advanced techniques for spore trapping, combined with quantitative PCR-based approaches, are becoming more prevalent for a range of airborne plant pathogens [41]. Preliminary data suggest that some of the primers developed for functional genes in this study are specific to *Itersonilia* and, hence, could be adapted for this purpose. Furthermore, the same approach may be applicable for the detection and quantification of the pathogen on seed, which would offer a more rapid alternative to the conventional five-day seed test.

## Figures and Tables

**Table 1:** Isolate number, host, and origin of *Itersonilia* spp. isolates characterised in this study. * indicates isolates where only DNA was available.

Culture	Fungal Species	Host Plant Material	Origin/Culture Collection	Country
IP1	*I. pastinacae*	Parsnip seed	Lincolnshire	England
IP2	*I. pastinacae*	Parsnip seed	Lincolnshire	England
IP3	*I. pastinacae*	Parsnip seed	Lincolnshire	England
IP4	*I. pastinacae*	Parsnip seed	NIAB	England
IP5	*I. pastinacae*	Parsnip seed	NIAB	England
IP6	*I. pastinacae*	Parsnip seed	NIAB	England
IP7	*I. pastinacae*	Parsnip seed	Crop Health Services, Fera	England
IP8	*I. pastinacae*	Parsnip seed	Crop Health Services, Fera	England
IP9	*I. pastinacae*	Parsnip seed	Crop Health Services, Fera	England
IP10	*I. pastinacae*	Parsnip seed	Heves	Hungary
IP11	*I. pastinacae*	Parsnip root	Stockbridge Technology Centre	England
IP12	*I. pastinacae*	Parsnip root	Stockbridge Technology Centre	England
IP13	*Itersonilia* sp.	Chrysanthemum petal	Stockbridge Technology Centre	England
IP14 *	*Itersonilia* sp.	Chrysanthemum petal	Stockbridge Technology Centre	England
IP15	*Itersonilia* sp.	Dill leaves	Stockbridge Technology Centre	England
IP16 *	*Itersonilia* sp.	Dill leaves	Stockbridge Technology Centre	England
IP17	*Itersonilia* sp.	Dill leaves	Stockbridge Technology Centre	England
IP18 *	*I. pastinacae*	Parsnip seed	Heves	Hungary
IP19	*I. pastinacae*	Parsnip seed	Heves	Hungary
IP20	*I. pastinacae*	Parsnip seed	Poituo-Charentes	France
IP21	*I. pastinacae*	Parsnip seed	Poituo-Charentes	France
IP22	*I. pastinacae*	Parsnip seed	Poituo-Charentes	France
IP23	*I. pastinacae*	Parsnip seed	Kent	England
IP24	*I. pastinacae*	Parsnip seed	Lincolnshire	England
IP25	*I. pastinacae*	Parsnip seed	Lincolnshire	England
IP26	*Itersonilia* sp.	Chrysanthemum petal	ADAS	England
IP27	*I. pastinacae*	Parsnip leaves	Norfolk	England
IP28	*I. pastinacae*	Parsnip leaves	ADAS	England
IP29	*I. pastinacae*	Parsnip root	Vegetable Consultancy Services	England
IP30	*I. pastinacae*	Parsnip root	Lincolnshire	England
IP31	*I. pastinacae*	Parsnip seed	Wanganui	New Zealand
IP32	*I. pastinacae*	Parsnip seed	Wanganui	New Zealand
IP33	*I. pastinacae*	Parsnip seed	Wanganui	New Zealand
IP34	*I. pastinacae*	Parsnip seed	Wanganui	New Zealand
IP35	*I. pastinacae*	Parsnip leaves	Nottingham	England
IP36	*Itersonilia* sp.	Fennel leaves	Middlesex	England
IP37	*Itersonilia* sp.	Dill leaves	Middlesex	England
IP38	*Itersonilia* sp.	Parsley leaves	Middlesex	England
IP39	*I. pastinacae*	Parsnip root	Cupar, Fife	Scotland
IP40	*I. pastinacae*	Parsnip root	Östersund	Sweden
IP41	*I. pastinacae*	Parsnip root	Nottingham	England
IP42	*I. pastinacae*	Parsnip seed	Poituo-Charentes	France
IP43	*I. pastinacae*	Parsnip seed	Poituo-Charentes	France
IP44	*I. pastinacae*	Parsnip seed	Poituo-Charentes	France
IP45	*I. pastinacae*	Parsnip seed	Poituo-Charentes	France
IP46	*I. pastinacae*	Parsnip seed	Poituo-Charentes	France
IP47	*I. pastinacae*	Parsnip seed	Poituo-Charentes	France
IP48	*I. pastinacae*	Parsnip seed	Poituo-Charentes	France
IP49	*I. pastinacae*	Parsnip seed	Poituo-Charentes	France
IP50	*I. pastinacae*	Parsnip seed	Poituo-Charentes	France
IP51	*I. pastinacae*	Parsnip seed	Poituo-Charentes	France

**Table 2:** Target gene loci, primers, and thermocycling conditions for PCR and sequencing of *Itersonilia* spp. isolates [26,27,28].

Genetic Locus		Primer Code	Primer Sequence (5′-3′)	Length (bp)	Thermocycling Conditions	Amplicon Size (bp)	Source
*ITS*	Nuclear rDNA Internal transcribed spacer regions	ITS1	TCCGTAGGTGAACCTGCGG	19	one cycle of 2 min at 94 °C, 40 cycles of 35 s at 94 °C, 55 s at 61 °C and 1 min at 72 °C, followed by one cycle of 10 min at 72 °C	444	[26]
		ITS4	TCCTCCGCTTATTGATATGC	20			[27]
*EF-1α*	Translation Elongation Factor 1-α	EF595F	CGTGACTTCATCAAGAACATG	21	one cycle of 2 min at 94 °C, 40 cycles of 35 s at 94 °C, 55 s at 61 °C and 1 min at 72 °C, followed by one cycle of 10 min at 72 °C	392	[28]
		EF1160R	CCGATCTTGTAGACGTCCTG	20			
*Rpb2*	RNA Polymerase II	IP RpbII F	GACTTTGACCTGACGCCCTCTC	22	one cycle of 2 min at 94 °C, 30 cycles of 30 s at 94 °C, 1 min at 68 °C and 1 min at 72 °C, followed by one cycle of 10 min at 72 °C	1186	Designed from Isolate IP10 Genome
	Second largest subunit	IP RpbII R	AAGGGCCGAGATTCAGTCAG	20			
*TUB2*	Partial β-Tubulin	TUB2 55F	GCGTAGCCGACCATGAAGAAGC	22	one cycle of 2 min at 94 °C, 35 cycles of 45 s at 94 °C, 30 s at 68 °C and 1 min at 72 °C, followed by one cycle of 7 min at 72 °C	559	Designed from Isolate IP10 Genome
		TUB2 536R	ACACGGTCGTCGAGCCCTACAA	21			
*LSU*	Partial 28s rRNA gene	IP LSU F	ATGCGAGTTTCTGCTATCCTGAG	23	one cycle of 2 min at 94 °C, 30 cycles of 30 s at 94 °C, 1 min at 59 °C and 1 min at 72 °C, followed by one cycle of 7 min at 72 °C	214	Designed from Isolate IP10 Genome
		IP LSU R	ATCAATAAGCGGAGGAAAAGAAAC	23			
*SSU*	Partial 18s rRNA gene	IP SSU F	CGTCAATTCCTTTAAGTTTCAGC	23	one cycle of 2 min at 94 °C, 30 cycles of 30 s at 94 °C, 1 min at 48 °C and 1 min at 72 °C, followed by one cycle of 7 min at 72 °C	21	Designed from Isolate IP10 Genome
		IP SSU R	TATCTGCCCTATCAACTTTC	20			
*TTF*	Triosephosphate Transporter Family	588 F	CCCCGGGCGCTGAGTAGG	18	one cycle of 2 min at 94 °C, 15 cycles of 30 s at 94 °C, 1 min at 71 °C (decreasing 1 °C per cycle) and 1 min at 72 °C, followed by 25 cycles of 30 s at 94 °C, 1 min at 70 °C and 1 min at 72 °C followed by one cycle of 7 min at 72 °C	422	Designed from Isolate IP10 Genome
		1159 R	TGAGGGAGTGCGAGAAGTGTTAGC	24			
*tMT*	tRNA methyl transferase	36 F	GACGGGACCGATCTGCGACTGCTC	24	one cycle of 2 min at 94 °C, 15 cycles of 30 s at 94 °C, 1 min at 71 °C (decreasing 1 °C per cycle) and 1 min at 72 °C, followed by 25 cycles of 30 s at 94 °C, 1 min at 70 °C and 1 min at 72 °C followed by one cycle of 7 min at 72 °C	612	Designed from Isolate IP10 Genome
		437 R	GCCGATGACCTGACGACCGCTGTG	24			
*CDH*	Cellubiose Dehydrogenase	6381 F	GCAGTTGGCGCAGGCTATG	19	one cycle of 2 min at 94 °C, 15 cycles of 30 s at 94 °C, 1 min at 70 °C (decreasing 1 °C per cycle) and 1 min at 72 °C, followed by 25 cycles of 30 s at 94 °C, 1 min at 69 °C and 1 min at 72 °C followed by one cycle of 7 min at 72 °C	610	Designed from Isolate IP10 Genome
		6927 R	AGGAGGCGTGAGAAGAGTGTGAGG	24			

## Data Availability

The datasets generated during this study are available from the corresponding author on reasonable request.
